# Within‐subject reliability of brain networks during advanced meditation: An intensively sampled 7 Tesla MRI case study

**DOI:** 10.1002/hbm.26666

**Published:** 2024-05-10

**Authors:** Saampras Ganesan, Winson F. Z. Yang, Avijit Chowdhury, Andrew Zalesky, Matthew D. Sacchet

**Affiliations:** ^1^ Department of Psychiatry Melbourne Neuropsychiatry Centre Carlton Victoria Australia; ^2^ Department of Biomedical Engineering The University of Melbourne Carlton Victoria Australia; ^3^ Contemplative Studies Centre, Melbourne School of Psychological Sciences The University of Melbourne Melbourne Victoria Australia; ^4^ Meditation Research Program, Department of Psychiatry, Massachusetts General Hospital Harvard Medical School Boston Massachusetts USA; ^5^ Athinoula A. Martinos Center for Biomedical Imaging, Department of Radiology, Massachusetts General Hospital Harvard Medical School Boston Massachusetts USA

**Keywords:** 7 T functional MRI, advanced meditation, consciousness, intraclass correlation (ICC), jhana, neurophenomenology, within‐subject reliability

## Abstract

Advanced meditation such as jhana meditation can produce various altered states of consciousness (jhanas) and cultivate rewarding psychological qualities including joy, peace, compassion, and attentional stability. Mapping the neurobiological substrates of jhana meditation can inform the development and application of advanced meditation to enhance well‐being. Only two prior studies have attempted to investigate the neural correlates of jhana meditation, and the rarity of adept practitioners has largely restricted the size and extent of these studies. Therefore, examining the consistency and reliability of observed brain responses associated with jhana meditation can be valuable. In this study, we aimed to characterize functional magnetic resonance imaging (fMRI) reliability within a single subject over repeated runs in canonical brain networks during jhana meditation performed by an adept practitioner over 5 days (27 fMRI runs) inside an ultra‐high field 7 Tesla MRI scanner. We found that thalamus and several cortical networks, that is, the somatomotor, limbic, default‐mode, control, and temporo‐parietal, demonstrated good within‐subject reliability across all jhanas. Additionally, we found that several other relevant brain networks (e.g., attention, salience) showed noticeable increases in reliability when fMRI measurements were adjusted for variability in self‐reported phenomenology related to jhana meditation. Overall, we present a preliminary template of reliable brain areas likely underpinning core neurocognitive elements of jhana meditation, and highlight the utility of neurophenomenological experimental designs for better characterizing neuronal variability associated with advanced meditative states.

## Graphical abstract

Graphical illustration of the most reliable brain areas associated with advanced and rare meditation states, called jhanas, determined from an intensively sampled 7 T functional magnetic resonance imaging (fMRI) dataset, using an approach of intraclass correlation modified for estimating within‐subject fMRI reliability at the level of canonical brain networks.

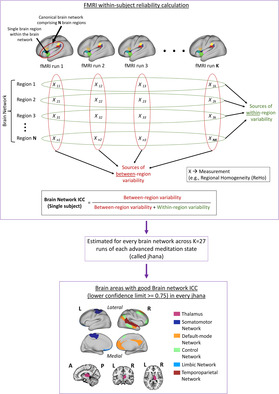

## 1 INTRODUCTION

The practice of meditation includes a wide range of simple and advanced attentional training techniques that can profoundly alter an individual's level of consciousness and degree of awareness (Laukkonen & Slagter, 2021; Timmermann et al., 2023), de‐reify perceptual processes (Giommi et al., 2023; Laukkonen & Slagter, 2021), and cultivate joy, tranquility, and compassion (Desbordes et al., 2014; Hölzel et al., 2011; Woods et al., 2023). Some of these benefits of meditation likely become stronger, more discernible, and longer‐lasting with meditation expertise, experience, and skilful practice (Dennison, 2019; Galante et al., 2023; Gunaratana, 1988; Hagerty et al., 2013; Laukkonen et al., 2023; Woods et al., 2023; Wright et al., 2023). Additionally, increasing dosage of meditation and mastery of advanced meditation can result in distinct milestones in practice (Galante et al., 2023), such as voluntary cessations of consciousness leading to lasting psychological insights and clarity (Chowdhury et al., 2023; Laukkonen et al., 2023). One advanced meditation technique introduced more than 2000 years ago is *jhana* (or *Dhyana* in Sanskrit) meditation (Gunaratana, 1988). This type of meditation involves sequential stages of highly absorptive and attentive states of mind called jhanas, which are facilitated by skilful progression in attentional quality, concentrative power, and sensory attenuation (Dennison, 2019; Laukkonen et al., 2023). Typically, jhana meditation is classified into eight progressively deeper meditative states called jhanas (Gunaratana, 1988).

The first four jhanas are termed “form” states. These states (i.e., J1, J2, J3, J4) involve focus on tangible objects grounded in sensory perception and are often accompanied by bliss/euphoria (Dennison, 2019; Gunaratana, 1988; Laukkonen et al., 2023). Among J1, J2, and J3, traversal from one jhana to the next typically involves shedding of some of the former jhana's undesirable phenomenological qualities. The subsequent jhanas (i.e., J5, J6, J7, J8) are called “formless states” since they are described as involving sensory perception and encompassing boundless experiences grounded in equanimity (Gunaratana, 1988; Laukkonen et al., 2023). Consequently, phenomenological sampling during “formless” states can be challenging. For further context and detailed phenomenological descriptions of each jhana, refer to work by Brasington (2015), Gunaratana (1988), Sayadaw (2010), and Shankman (2008b). Systematic investigation of jhana meditation using high‐resolution neuroimaging like functional magnetic resonance imaging (fMRI) can enable insights into the neural mechanisms underlying the different jhanas and complement existing phenomenological characterizations of meditative stages (Sparby & Sacchet, 2022). The therapeutic utility and accessibility of jhana meditation can hence be potentially enhanced. However, to our knowledge, only two studies have thus far explored the neurobiological substrates of jhana meditation using fMRI, one conducted by Hagerty et al. (2013), and another by our research team (Yang et al., 2023).

Relative to rest, Hagerty et al. (2013) found that jhana meditation can influence fMRI activity in brain regions associated with sensory processing (e.g., visual and auditory regions), attentional monitoring (e.g., anterior cingulate cortex [ACC]), and reward processing (e.g., medial orbitofrontal cortex [OFC], nucleus accumbens [NAc]). Yang et al. (2023) found that specific jhanas can influence fMRI responses in multiple other regions, namely insula, medial prefrontal cortex (mPFC), dorsolateral prefrontal cortex (DLPFC), cingulate areas, hippocampus, somatomotor areas, thalamus, brainstem, and cerebellum. Many of these brain regions also constitute canonical brain networks widely implicated in self‐referential processing (default‐mode network), awareness of bodily sensations (salience network), and attentional control (control network) during other meditation techniques involving focused attention (Ganesan, Beyer, et al., 2022; Sezer et al., 2022). Some of these brain systems may underpin key phenomenological elements and alterations within jhana meditation, including sensory deprivation, one‐pointed concentration, euphoria, and equanimity (Yang et al., 2023).

The sparsity of fMRI literature on jhana meditation may be due, in part, to limited availability of highly skilled meditation experts who can reliably evoke distinct jhanas volitionally and repeatably under experimentally controlled conditions (Dennison, 2019; Hagerty et al., 2013; Yang et al., 2023). The high proficiency necessary to evoke such complex and rare mental states thus highlight the utility of longitudinal single‐subject study designs for brain mapping of advanced meditation. Such study designs involve repeatedly scanning a single eligible and willing subject under similar experimental conditions over many days, months, or years (Kajimura et al., 2020; Poldrack et al., 2015; Yang et al., 2023). With the advent of ultra‐high field 7 Tesla fMRI, the reliability and quality of single‐subject longitudinal case‐studies for complex tasks such as advanced meditation can be bolstered, as 7 Tesla fMRI offers superior neuroanatomical resolution, statistical power, and signal quality across the whole brain compared to its counterparts (i.e., 3 Tesla or 1.5 Tesla) (Hale et al., 2010; Pohmann et al., 2016; Torrisi et al., 2018; Trattnig et al., 2018; Viessmann & Polimeni, 2021). Although single‐subject experimental designs do not provide insight into generalizability of findings, they permit dense longitudinal phenotyping that uniquely enables systematic examination of dynamics and consistency of brain responses over time under repeated presentations of similar task demands or conditions (Poldrack et al., 2015).

Consistency (or reliability) of fMRI responses under repeated measurements can be quantified using intraclass correlation coefficients (ICC) (G. Chen et al., 2018; Noble et al., 2021). Typically, ICC is computed as the proportion of total measured variance in fMRI responses that belongs to variability between subjects in multi‐subject study designs. Recent meta‐analytic evidence found that reliability in the context of human fMRI literature has generally been low, especially with functional connectivity (Noble et al., 2019) and task‐fMRI measures (Elliott et al., 2020). Therefore, given the complexity associated with jhana meditation, reliability assessments can be invaluable to examine whether it is feasible to replicate complex states of consciousness (jhanas) inside an MRI scanner. Without estimates of reliability, the practical utility of an observed correspondence (validity) between a specific brain network and jhana would remain limited (Bennett & Miller, 2010; Noble et al., 2019), thus constraining the scope of neurobiological inferences associated with advanced meditation. Therefore, assessing reliability (e.g., ICC estimates of regional homogeneity [ReHo] values) in addition to functional relevance (e.g., ReHo values) of brain areas can be instrumental for precise and robust functional brain mapping of jhana meditation.

Given the limited and small neuroimaging literature in jhana meditation, it can be challenging to establish the most consistent and reliable brain responses associated with jhanas, without an available approach for within‐subject fMRI reliability assessments. So far, ICC has largely been applied in the context of multi‐subject study designs to measure reliability (G. Chen et al., 2018; Noble et al., 2021), thus highlighting the need for modified ICC implementations that facilitate reliability assessments in single‐subject fMRI studies. ICC can be calculated from a variety of fMRI measures including percent signal change, *t*‐statistics, beta coefficients, connectivity, and ReHo (Noble et al., 2021).

ReHo is a measure of functional synchronization between voxels and their neighbors (Zang et al., 2004), reflecting the extent of local functional integration and underlying activity among brain areas (Jiang & Zuo, 2016). ReHo values can thereby illuminate the most important functional hubs within whole‐brain functional connectomes associated with specific mental states (Jiang & Zuo, 2016). Compared to several other fMRI indices, ReHo has shown superior reproducibility and stability across repeated measurements in resting‐state fMRI, due to robustness to non‐neural intraindividual variability (X. Chen et al., 2018; Li et al., 2012; Zuo & Xing, 2014). Consequently, mapping ReHo of distinct jhanas can illuminate crucial hubs of brain regions and networks that are more likely associated with subtle phenomenological elements of rare consciousness states, and less influenced by artifacts and other non‐specific effects. Importantly, identifying brain areas with highly stable ReHo values across repeated measurements of jhanas can further highlight the most reliable and replicable hubs of neurobiology associated with each jhana, which may underpin certain core characteristics of jhana phenomenology. In other words, maps of ReHo reliability hotspots could potentially serve as brain templates and priors that can improve statistical power of future neuroimaging studies of jhana meditation, especially considering the infancy of jhana neuroimaging. Brain networks with high reliability estimates in specific jhanas imply stable associations with specific qualities of those jhanas, which can thereby improve the statistical likelihood of detecting effects in these a priori‐defined networks with small samples (Zuo et al., 2019) prevalent in neuroimaging of rare states. While previous work using the single‐subject 7 T fMRI dataset by Yang et al. (2023) has characterized a comprehensive preliminary brain map of relevant brain areas associated with the eight jhanas and their phenomenology, the current work specifically utilizes the aspect of intensive sampling in the dataset to evaluate reliability and consistency of the observed brain responses when distinct jhanas are repeatedly evoked over time.

Our primary aim was to evaluate the reliability of ReHo at the level of canonical brain networks and groups across the whole brain in each jhana, using a modified ICC implementation suitable for within‐subject longitudinal studies. We hypothesized that various brain networks and areas, including those previously implicated in jhana meditation (i.e., default‐mode network, salience network, control network, thalamus, OFC, cerebellum, and brainstem), would demonstrate high reliability during different jhanas. Since fMRI activity and ReHo in specific brain areas can exhibit distinct patterns of change across jhanas (Hagerty et al., 2013; Yang et al., 2023), we additionally explored how reliability differed between jhanas.

Focused attention is a foundational element of jhana meditation (Gunaratana, 1988), and attentional qualities can influence ReHo within insula, DLPFC, mPFC, OFC, thalamus, hippocampus, and visual cortex (Yang et al., 2023). Incorporating subjective phenomenological measures into neuroimaging models can potentially improve explanatory and statistical power (Timmermann et al., 2023). Therefore, our second aim was to examine how and whether controlling for relevant phenomenological measures may impact our findings. We hypothesized that controlling for phenomenology prior to ICC calculations would improve within‐subject ReHo reliability.

## 2 METHODOLOGY

### 2.1 Data characteristics

#### 2.1.1 Case‐study subject

This case study involved one adept meditator (age = 51 years) extensively trained in jhana meditation, with 26 years of cumulative lifetime meditation experience (estimated daily and retreat practice of 23,000 hours) (refer to Yang et al. (2023) for further details). The subject provided informed consent, and did not meet criteria for any neuropsychiatric (measured through Mini‐International Neuropsychiatric Interview; Sheehan et al., 1998) or cognitive (measured through Mini‐Mental State Examination; Folstein et al., 1975) impairments. The study was approved by the Mass General Brigham IRB.

#### 2.1.2 MRI acquisition

Whole‐brain fMRI BOLD data was acquired from the case‐study subject (*N* = 1) on 5 consecutive days using 7 Tesla MRI scanner (Siemens Magnetom Terra) with a 32‐channel head coil (repetition time [TR] = 2.9 s, echo time [TE] = 30 ms, flip angle = 75°, field of view [FOV] = 189 × 255 mm, parallel imaging GRAPPA factor = 3, isotropic voxel size = 1.1 mm, 126 slices). Concordant physiological signals (i.e., cardiac activity using pulse oximetry and respiration using breathing bellows) were also acquired during fMRI scanning. Whole‐brain T1‐weighted structural images were acquired with TR = 2.53 s, TE = 1.65 ms, flip angle = 7°, isotropic voxel size = 0.8 mm, FOV = 240 × 240 mm, and GRAPPA factor = 2.

Further details on data acquisition parameters and complete experimental design have been presented elsewhere (see Yang et al., 2023). Briefly, the subject was scanned during different tasks which included jhana meditation with their eyes closed. For our analyses here, we included the fMRI runs that were recorded specifically during jhana meditation (i.e., *k* = 27 fMRI runs). Within each such fMRI run (jhana meditation duration = 512 ± 127 s), sequential jhanas (J1 to J5) were segmented based on self‐reported transitions (button presses). Note that transitions involving “formless” jhanas J6 to J8 could not be reported since the process of self‐reporting is typically antagonistic to the progression of these advanced absorptive meditative states. Consequently, for analyses reported hereafter, data from J6 to J8 were merged as one advanced jhana J6‐J8.

#### 2.1.3 Self‐reported phenomenological ratings

After every fMRI run of jhana meditation, the subject provided ratings from 1 to 10 for various phenomenological aspects of their meditation in the MRI scanner. Out of the different phenomenological items examined across the different jhanas, “stability of attention,” “width of attention,” and “intensity of jhana” were considered for the current analyses. These measures aimed to capture the same phenomenological quality consistently and comparably across all the jhanas, unlike the other excluded phenomenological measures that were distinct to specific jhanas. Consequently, the impact of phenomenology on ReHo reliability could be examined across all the jhanas equivalently, and also compared between jhanas.

“Stability of attention” ratings were expected to capture the tranquility and non‐volatility of focused attention during each jhana. “Width of attention” ratings were expected to indicate the scope of attentional focus, that is, laser‐like narrow focused attention (rated lower) or broad focused attention encompassing wider attentional fields (rated higher). “Intensity of jhana” ratings were meant to capture the intensity of each jhana's characteristic phenomenology (more intense was rated higher). Further details regarding other general and state‐specific measures that were recorded are presented elsewhere (Yang et al., 2023).

### 2.2 Data analysis

#### 2.2.1 fMRI preprocessing

fMRI preprocessing was performed using AFNI toolbox. A detailed description of the preprocessing steps has been provided in Yang et al. (2023). Briefly, the preprocessing pipeline included skull‐stripping, bias‐field correction, de‐spiking, physiological artifact correction using RETROspective Image CORrection (RETROICOR; Glover et al., 2000; Model predictors—four cardiac, four respiration, one respiration volume per time [Birn et al., 2008], and one heart rate [Chang et al., 2009]), slice‐timing correction, magnetic field inhomogeneity distortion correction, head‐motion correction (six standard motion parameters), fMRI volume scrubbing based on head motion (>0.3 mm frame‐wise displacement and 5% outlier voxels), non‐linear registration between functional, structural, and template (MNI152_2009) brain images, band‐pass filtering (0.01–0.1 Hz), and regression of average cerebrospinal fluid signal.

#### 2.2.2 ReHo analysis

ReHo refers to the temporal coherence of BOLD timeseries of a voxel and its neighboring voxels, that is, local functional connectivity (Zang et al., 2004). While larger ReHo values indicate greater homogeneity and functional integration of BOLD responses within a defined brain area/region, smaller ReHo values suggest greater functional segregation (Jiang & Zuo, 2016).

ReHo was estimated through Kendall's coefficient of concordance (KCC), that is, an index of similarity between BOLD timeseries of multiple neighboring voxels. Whole‐brain voxel‐wise KCC was calculated for each voxel with 26 neighboring voxels (within 3 × 3 × 3 voxels cube space). Consequently, voxel‐wise ReHo values were generated for every segmented jhana within each fMRI run and transformed into normalized *z*‐scores. The normalized ReHo maps were then smoothed using a 6 mm full‐width half‐maximum Gaussian kernel. Subsequently, the voxel‐wise smoothed and normalized ReHo values were averaged within distinct regions of interest (ROIs) defined by standard brain atlases that excluded white matter and ventricular brain areas. Specifically, the Schaefer‐400 cortex atlas was used to parcellate cortical areas (400 ROIs) (Schaefer et al., 2018), the Melbourne S4‐subcortex atlas for subcortical regions (62 ROIs) (Tian et al., 2020), the Bianciardi brainstem atlas for brainstem (66 ROIs) (Bianciardi et al., 2016), and the Multi‐Domain Task Battery (MDTB) functional cerebellar atlas for the cerebellum (10 ROIs) (King et al., 2019).

Thus, the whole brain was parcellated into 538 distinct ROIs, yielding an average ReHo value for each ROI. All cortical ROIs were grouped into 34 canonical unilateral brain networks (i.e., 17 networks from each hemisphere), following the well‐established functional parcellation scheme of Yeo et al. (2011). Subcortical (eight unilateral groups), cerebellum (1 group), and brainstem (7 groups) ROIs were grouped based on function and anatomical proximity such that each group contained at least five regions. For instance, all hippocampal and thalamic subregions from each hemisphere were grouped based on functional relatedness into left or right “hippocampus” and “thalamus,” respectively. On the other hand, caudate and putamen subregions from each hemisphere were combined based on anatomical proximity to form left or right “Caudate and putamen” groups with more than five constituent subregions. The complete list of regions in each group/network is shown in Table S1 in Data S1. Note that “networks” here refers to the canonical cortical networks, while “groups” refers to the groupings of subcortical, brainstem and cerebellar subregions. Overall, the 538 ROIs/regions were grouped into 50 brain networks/groups for subsequent reliability analyses.

#### 2.2.3 Reliability using brain network intraclass correlation coefficient (brain network‐ICC)

To quantify the reliability of ReHo values associated with each jhana under repeated fMRI measurements in a single subject, we modified the commonly used ICC approach. A brief background on the traditional ICC approach used in multi‐subject studies can be found in Supplementary section SS1 in Data S1. ICC values vary between 0 and 1, and can be qualitatively classified as “poor” (ICC < 0.5), “moderate” (0.5–0.75), “good” (0.75–0.9), and “excellent” (ICC > 0.9) (Koo & Li, 2016; Liljequist et al., 2019).

**FIGURE 1:**
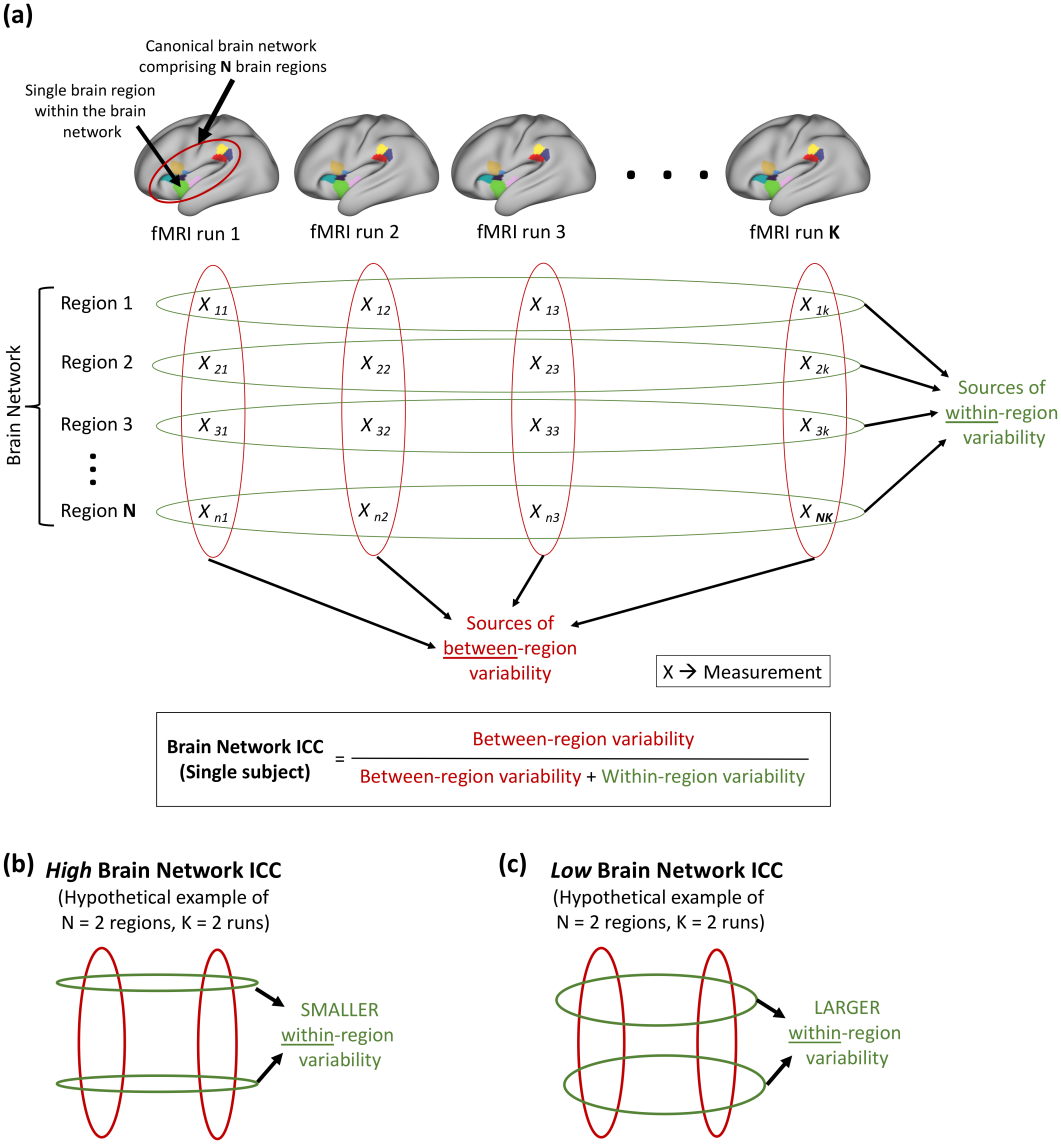
Graphical illustration of the proposed approach of estimating brain network intraclass correlation coefficient (ICC). (a) For a canonical brain network comprising *N* regions (indicated by different colors on the brain) from a single subject, brain network ICC can be used to estimate within‐subject reliability of fMRI measurements (*X*) across *K* repeated fMRI runs measuring a given cognitive state. Each column represents measurements from a specific fMRI run, and each overlaid red oval represents variability between regions within the run. On the other hand, each row represents repeated measurements corresponding to a specific region within the brain network, and each overlaid green oval represents variability within the region (or between runs). Brain network ICC is simply the proportion of total variability attributed to between‐region variability and assumes values between 0 and 1. (b) A hypothetical brain network with high brain network ICC (closer to 1), demonstrating high within‐subject reliability of fMRI measurements across *K* runs. Narrower green ovals indicate smaller variability within regions/between runs, and broader red ovals indicate larger variability between regions of a brain network. (c) A hypothetical brain network with low brain network ICC (closer to 0), demonstrating poor within‐subject reliability of fMRI measurements across *K* runs. Broader green ovals indicate larger within‐region/between‐run variability, and the smaller red ovals indicate comparatively smaller between‐region variability. fMRI, functional magnetic resonance imaging.

The current case‐study involves a single subject measured 27 times (i.e., 27 fMRI runs). Therefore, we modified the traditional ICC approach to enable evaluation of ReHo reliability within a single subject. We refer to this modified ICC approach as brain network ICC (Figure 1). For each jhana and each of the 50 brain networks/groups, we estimated brain network ICC by calculating the proportion of total variance of ReHo (across runs and regions/ROIs) that is attributed to variance of ReHo between regions/ROIs constituting the brain network/group (Figure 1a). Consequently, high brain network ICC would indicate that a larger proportion of the total variability is attributed to variability between regions within a brain network, and that variability within regions (i.e., between fMRI runs) is comparatively smaller (i.e., more reliable) (Figure 1b). A brain network/group with low ICC would imply larger within‐region variance (or larger variability between fMRI runs) compared to between‐region variance (Figure 1c). The network‐level scope of brain network ICC enabled within‐subject ICC calculations, wherein the reliability of each brain network/group across fMRI runs was evaluated independently.

We estimated brain network ICC in MATLAB (Salarian, 2023) using the standard mathematical formulation of ICC(A,1) (also referred to as ICC(2,1)) with two‐way mixed effects *model*, “absolute agreement” *type*, and “single‐rater” *definition* (G. Chen et al., 2018; Liljequist et al., 2019). These parameters were found to be most suitable for the proposed brain network ICC implementation, as elaborated in Supplementary section SS2 in Data S1.

For each jhana (J1–J5 and merged J6–J8) and each brain network/group, we estimated brain network ICC from the normalized ReHo values of constituent ROIs. We also computed 95% confidence interval limits for each estimated brain network ICC through bootstrapping of runs (5000 permutations). Specifically, for each region within a network/group, the 27 fMRI runs were randomly sampled with replacement. Brain network ICC was subsequently estimated from the ReHo of all regions with resampled fMRI runs within each network/group. For each network/group, this random resampling and ICC estimation were repeated 5000 times to determine the 2.5th and 97.5th percentiles of the ICC estimates (lower and upper limits of the 95% confidence interval). We then thresholded the brain networks/groups at “good” brain network ICC lower confidence limits (≥0.75) for each jhana separately. Note that brain network ICC involves single‐subject fMRI data. Hence, its range of values and choice of thresholds cannot be directly compared to that of traditional group‐level fMRI ICC which are generally lower (Elliott et al., 2020; Noble et al., 2019).

Incorporating relevant explanatory variables during fMRI reliability assessments can help adequately account for confounding effects that may be influencing fMRI signal and subsequent reliability estimates (G. Chen et al., 2018). Therefore, to further evaluate the impact of between‐run variability in self‐reported phenomenological measures (i.e., “stability of attention,” “width of attention,” and “intensity of jhana”) on ReHo reliability, we repeated the brain network ICC computations after controlling for these run‐wise self‐report measures. Specifically, we computed brain network ICC for each brain network/group using the residual ReHo of linear regressions involving the three self‐report measures. Although there were several other self‐report measures originally acquired in the study (see Yang et al., 2023 for details), only three of those measures, as mentioned above, captured the same phenomenological quality consistently and comparably across all the jhanas. The remaining measures were excluded from the current study since they were used to assess phenomenology specific to certain individual jhanas or groups of jhanas. Excluding these measures enabled the number and nature of covariates to be consistent for every jhana, thus promoting interpretability of findings and evaluation of differences in reliability between distinct jhanas.

Finally, we also repeated the brain network ICC computations after including the mean framewise displacement (mFD; 1 value per run in each jhana) as an additional covariate to further elucidate the influence of average head motion on ReHo reliability across runs.

## 3 RESULTS

For each jhana, within‐subject brain network ICC estimates were computed from region‐level ReHo values for each of 50 unilateral brain networks/groups spanning all cortical, subcortical, cerebellar, and brain stem areas. Subsequently, the lower limit of the 95% confidence interval of each ICC estimate was thresholded at “good” reliability (lower limit ICC ≥ 0.75). Several functional networks/groups within the cortical and subcortical areas showed above‐threshold reliability within each jhana, with commonalities and distinctions across the jhanas (see Figure S1 in Data S1 for brain maps of reliability within each individual jhana, and Figure S2 in Data S1 for the corresponding ReHo maps). Controlling for phenomenological variability (i.e., attentional stability, attentional width, and intensity of jhana) between runs led to improvements in reliability within each jhana.

We found no evidence of significant correlation between the ICC estimates (or their lower confidence limits) and number of regions (*N*) within the brain networks/groups in any jhana before and after controlling for phenomenological variability. This suggests that the ICC values were likely not influenced by the size of brain networks/groups. Similarly, the correlation between the brain network ICC estimates and average relative standard deviation of normalized ReHo values across constituent regions of brain networks (measuring between‐region variability) was nonsignificant across jhanas, suggesting minimal or no influence of within‐network ReHo variability on ICC estimates.

### 3.1 Common brain networks/groups with above‐threshold reliability in *every* jhana

**FIGURE 2:**
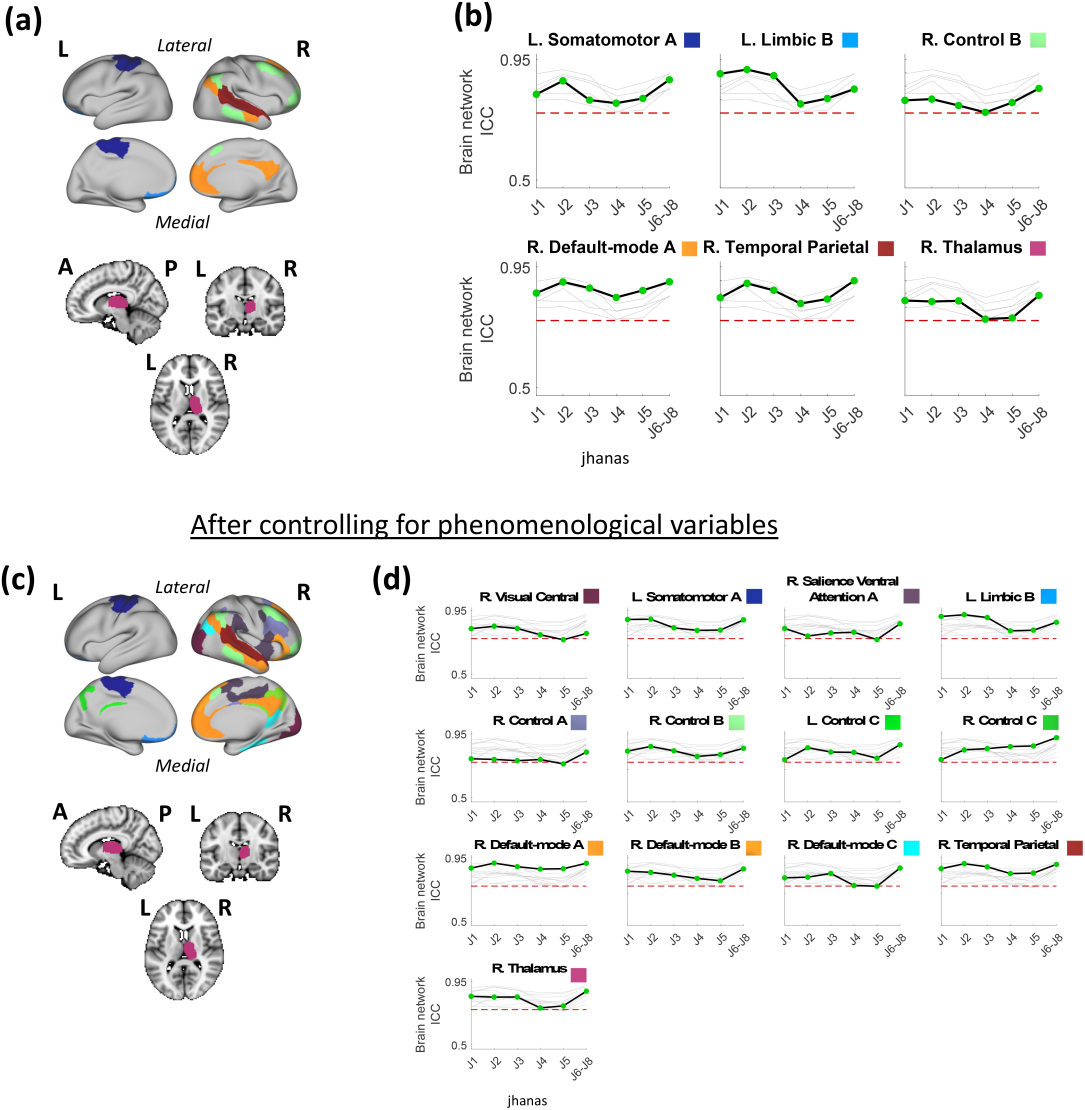
Brain surface (cortex) and volume (subcortex) visualizations of brain networks/groups with above‐threshold reliability across jhanas J1–J8, alongside graphical representations of changes in the lower confidence limits of their brain network ICC values across the sequential jhanas. Note that jhanas J6–J8 were merged as one advanced state. (a) The six brain networks/groups with above‐threshold reliability (ICC lower confidence limit ≥0.75) across jhanas J1 to J8 have been shown on the brain using distinct colors. Each color is labeled in panel (b). (b) Change in the lower confidence limit of brain network ICC across jhanas in each of the six networks/groups relative to one another, where ICC change of only the labeled network/group is highlighted (black line) in each graph while the remaining networks/groups are grayed out in the background. The network/group label above each graph is accompanied by its respective color as shown on the brain map in panel (a). The *y*‐axis of each graph represents brain network ICC values while the distinct jhanas (J1 to J6–J8) are marked on the *x*‐axes. (c) Similar to (a) showing 13 common brain networks/groups but after controlling the region‐level ReHo values for key self‐report phenomenological variables (1 value per fMRI run per jhana) including “Attentional stability,” “Attentional width,” and “jhana intensity.” (d) Similar to (b) after controlling the region‐level ReHo values for the self‐report phenomenological variables. The network/group label above each graph is accompanied by its respective color as shown on the brain map in panel (c). The dotted red line in each graph (in (b) and (d)) represents the threshold of “good” brain network ICC (i.e., ICC lower confidence limit of 0.75). A, anterior; fMRI, functional magnetic resonance imaging; ICC, intraclass coefficient correlation; L, left; P, posterior; R, right; ReHo, regional homogeneity.

Some of the brain networks/groups demonstrated above‐threshold reliability in every jhana (J1–J8) (Figure 2). Specifically, right thalamus, left somatomotor network A, right default‐mode network A, right control network B, left limbic network B, and right temporal parietal network showed above‐threshold brain network ICC estimates across all the jhanas (Figure 2a,b). Additionally, the average ReHo of the right thalamus and right temporal parietal network was consistently modest (ReHo ≥ 0.1) across all jhanas (Figure S2A in Data S1). After controlling for between‐run phenomenological variability, there were several additional networks/groups that showed above‐threshold brain network ICC estimates across all the jhanas, that is, right central visual network, right salience ventral attention network A, right control network A, bilateral control network C, right default‐mode network B, and right default‐mode network C (Figure 2c,d). The average ReHo of left and right control network C was consistently modest (ReHo ≥ 0.1) across all jhanas (Figure S2B in Data S1). Additionally controlling for between‐run mFD variability did not produce any new networks/groups that crossed the threshold in all the jhanas (Figure S3 in Data S1). However, brain network ICC of the right salience ventral attention network A dipped slightly below the threshold in jhana J5 alone (Figure S4 in Data S1).

### 3.2 Brain networks/groups with substantial variation in reliability across jhanas

**FIGURE 3:**
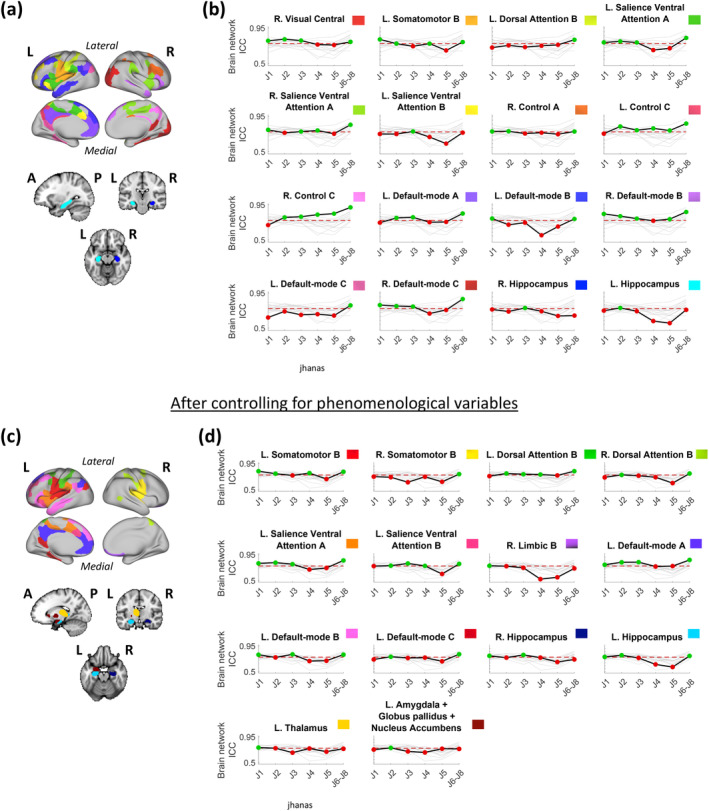
Brain surface (cortex) and volume (subcortex) visualizations of brain networks/groups with above‐threshold reliability in at most five of the six jhanas (where jhanas J6–J8 are merged). Changes in the lower confidence limits of brain network ICC across the jhanas in each of these distinct networks/groups are depicted graphically alongside their brain maps. (a) The 16 brain networks/groups with above‐threshold reliability (ICC lower confidence limit ≥0.75) in at most five jhanas have been shown on the brain using distinct colors labeled in panel (b). (b) Change in the lower confidence limit of brain network ICC across jhanas in each of the 16 networks/groups relative to one another, where ICC change of only the labeled network/group is highlighted (black line) in each graph while the remaining networks/groups are grayed out in the background. The network/group label above each graph is accompanied by its respective color as shown on the brain map in panel (a). The *y*‐axis of each graph represents brain network ICC values while the distinct jhanas (J1 to J6–J8) are marked on the *x*‐axes. (c) Similar to (a) showing 14 brain networks/groups after controlling the region‐level ReHo values for key self‐report phenomenological variables (1 value per fMRI run per jhana) including “Attentional stability,” “Attentional width,” and “jhana intensity.” (d) Similar to (b) after controlling the region‐level ReHo values for the same self‐report phenomenological variables. The network/group label above each graph is accompanied by its respective color as shown on the brain map in panel (c). In each graph of (b) and (d), the dotted red line represents the threshold of “good” brain network ICC (i.e., ICC lower confidence limit of 0.75). The green colored points indicate above‐threshold ICC (i.e., ICC lower confidence limit ≥0.75) while red colored points indicate below‐threshold ICC (i.e., ICC lower confidence limit <0.75) for a given jhana. A, anterior; fMRI, functional magnetic resonance imaging; ICC, intraclass coefficient correlation; L, left; P, posterior; R, right; ReHo, regional homogeneity.

Although some brain networks/groups showed above‐threshold ReHo reliability in all jhanas, the reliability of most networks/groups varied across jhanas. Figure 3 demonstrates all such networks/groups with above‐threshold ICC in at least one but not all jhanas.

Some networks/groups shared common patterns of change in reliability across multiple jhanas. Specifically, right central visual, right default‐mode C, and left salience ventral attention A networks showed above‐threshold reliability in jhanas J1, J2, J3, and J6–J8; bilateral control C networks showed above‐threshold reliability in all jhanas but J1; right hippocampus and left salience ventral attention network B showed above‐threshold ICC in J3 only; and left dorsal attention B and left default‐mode C network showed above‐threshold reliability in J6–J8 only (Figure 3b). On the other hand, several networks/groups also exhibited distinct patterns of change in reliability across jhanas. Particularly, left somatomotor network B had above‐threshold ICC in J1, J2, J4, and J6–J8; right salience ventral attention network A showed above‐threshold reliability in J1, J3, J4, and J6–J8; left default‐mode network A had above‐threshold ICC in J2, J3, and J6–J8; right control network A showed above‐threshold ICC in J1, J2, and J6–J8; left default‐mode network B had above‐threshold ICC in J1 and J6–J8; and left hippocampus showed above‐threshold reliability in J2 only (Figure 3b).

After controlling for between‐run (or within‐region) phenomenological variability, most networks/groups showed an increase in brain network ICC, with some crossing the threshold in additional jhanas. For instance, left dorsal attention network B crossed the threshold in J2, J3, and J4, left salience ventral attention network B crossed the threshold in J2, J4, and J6–J8, left default‐mode network A crossed the threshold in J1, left default‐mode network B crossed the threshold in J3, left default‐mode network C crossed the threshold in J2, right hippocampus crossed the threshold in J1, and left hippocampus crossed the threshold in J1 and J6–J8 (Figure 2d vs. Figure 2b).

Few of the networks/groups crossed the reliability threshold (above‐threshold) for the first time in any jhana, only after controlling for phenomenological variables. These networks/groups include right somatomotor network B (above‐threshold in J6–J8), right dorsal attention network B (above‐threshold in J2 and J6–J8), right limbic network B (above‐threshold in J1), left thalamus (above‐threshold in J1), and left amygdala + globus pallidus + nucleus accumbens (above‐threshold in J2) (Figure 3d). In other words, the reliability of these networks/groups was below‐threshold in all jhanas, prior to accounting for phenomenological variability.

Complete individual brain maps of above‐threshold reliability associated with each jhana before and after phenomenological control can be found in Figure S1 in Data S1, and their corresponding average ReHo maps are shown in Figure S2 in Data S1. Note that several networks with above‐threshold reliability after phenomenological control also had modest average ReHo (ReHo ≥ 0.1) in specific jhanas (e.g., right control network A in J1, J2, J3, J4, and J6–J8; right control network B in J1, J2, J3, and J4; left default‐mode network B in J1, J3, and J6–J8; left salience ventral attention network B in J2, J3, J4, and J6–J8; left default‐mode network C in J2 and J6–J8; left default‐mode network A in J3 and J6–J8; and right hippocampus in J3).

Inclusion of mFD as an additional covariate led to an overall increase in brain network ICC among most networks/groups. However, following mFD regression, only two cortical networks surpassed the reliability threshold for the first time in any jhana (J2 and J6–J8), that is, left limbic network A and left control network B. Similarly, only the subcortical groups (left thalamus and left amygdala + globus pallidus +nucleus accumbens) surpassed the threshold in two additional jhanas, while the cortical groups surpassed the threshold in only one additional jhana (Figure S4 in Data S1) following mFD regression.

## 4 DISCUSSION

We investigated within‐subject reliability of ReHo among brain regions constituting distinct canonical brain networks associated with different states of jhana meditation in a single adept meditator (*N* = 1) using ultrahigh field 7 Tesla fMRI. To accomplish this, we computed brain network ICC, a modified approach of ICC for within‐subject longitudinal fMRI designs, from ReHo estimates across the whole brain for each demarcated jhana measured 27 times (*k* = 27) over 5 days. This is the first study to examine fMRI within‐subject reliability associated with distinct jhanas that were intensively sampled.

We found several cortical networks and subcortical areas that demonstrated good (i.e., above‐threshold) reliability (lower confidence limit ICC ≥0.75) of ReHo in different jhanas. As hypothesized, many of the key brain areas previously implicated in jhana meditation showed above‐threshold reliability in all jhanas, including primary somatomotor areas (somatomotor network), OFC (limbic network), lateral PFC and DLPFC areas (control network), mPFC and PCC (default‐mode network), temporoparietal areas (temporal parietal network), and thalamus. Notably, average ReHo values of the thalamus and temporal parietal network were also modest (ReHo ≥ 0.1) in all the jhanas. There were also several networks/groups that exhibited good reliability in specific but not all jhanas. Furthermore, accounting for inter‐run variability in self‐reported phenomenology (i.e., attention and depth of jhanas) increased overall reliability, with several additional networks/groups crossing the reliability threshold (i.e., visual, salience, attentional, and more control and default‐mode areas). The average ReHo of these areas was also consistently modest across multiple jhanas. Although additionally controlling for inter‐run variability in head motion (mFD) also increased overall reliability across all networks/groups, it did not uniquely affect the reliability of specific brain networks/groups as much.

Our findings hence identify some of the most reliable brain areas pertinent to jhana meditation, which can subsequently facilitate more rigorous and precise neuroimaging investigations of jhana meditation as well as advanced meditation more broadly. Controlling for variability in phenomenology relevant to jhana meditation improved reliability estimates, thereby highlighting the utility of phenomenological sampling to meaningfully explain additional neurobiological variance, which is potentially associated with subtle fluctuations in actual mental states but typically disregarded as noise. This demonstrates the importance of incorporating neurophenomenology in the investigation of advanced meditation practices, as well as contemplative and psychological research broadly. Furthermore, variability in head motion demonstrated minimal impact on the reliability estimates, thus implying that the reliabilities of specific networks/groups were less likely driven by such artifacts.

Broadly, this study demonstrates that advanced meditation states that are rarely studied in science can indeed be investigated reliably and rigorously given the appropriate technology and methodology. The combination of ultra‐high field MRI, intensive neurophenomenological sampling, brain network ICC, and an advanced meditation technique with defined sequential stages (i.e., jhana meditation) makes the scientific investigation of complex states of consciousness and their reliability more amenable.

### 4.1 Brain areas showing most reliable engagement with every jhana

Brain networks/groups demonstrating good ReHo reliability across every jhana mainly comprise supplementary motor area (SMA) and primary somatomotor cortex (somatomotor network), OFC (limbic network), inferior parietal lobe, lateral PFC and DLPFC areas (control network), mPFC, PCC and precuneus regions (default‐mode network), superior temporal gyri (STG) and temporo‐parietal junction (TPJ) (temporal parietal network), and thalamus. Due to their good replicability across jhanas, consistent implication in previous neuroimaging investigations of jhana meditation (Dennison, 2019; Hagerty et al., 2013; Yang et al., 2023) as well as general functional relevance to various elements of jhana meditation, some of these brain networks and regions likely underpin core characteristics of jhanas.

For instance, it has been shown that jhanas attenuate activity in brain networks relevant to higher‐order thinking and mental processing, including default‐mode, somatomotor, and frontal (Hagerty et al., 2013). In the current study, we found that these same areas also demonstrate good ReHo reliability across several repeated instances of jhana meditation. Similarly, OFC, another region with good within‐subject reliability, has also been previously implicated in jhana meditation for its role in promoting jhanic pleasure and euphoria (Hagerty et al., 2013; Yang et al., 2023). Therefore, observations of functional validity (from previous literature) coupled with good reliability (from the current study) highlight the relevance of these brain areas in subserving fundamental and replicable attributes of jhana meditation.

Specifically, attaining jhanas requires highly focused attention, which is typically antagonistic to the default‐mode of mental processing and awareness (Dennison, 2019; Ganesan, Beyer, et al., 2022; Ganesan et al., 2023; Hagerty et al., 2013; Laukkonen & Slagter, 2021; Yang et al., 2023). Consequently, jhana meditation may robustly influence BOLD responses within default‐mode regions (e.g., PCC, mPFC, precuneus) involved in self‐referential processing and thought (Raichle, 2015), within somatomotor areas (e.g., SMA) implicated in internal speech processing (Hertrich et al., 2016; Kim, 2012; Summerfield et al., 2009), and in other similar areas (e.g., STG within temporal parietal network [Chang et al., 2010; Shergill et al., 2002]). Similarly, OFC (within the limbic network) is widely associated with reward‐processing, valuation (Knudsen & Wallis, 2022; Kringelbach, 2005), and mood (Rudebeck & Rich, 2018), which may consistently contribute toward subjective processing of euphoria, bodily pleasure, and equanimity associated with jhana meditation. Although the average ReHo of some of these networks may be low due to functional specialization and heterogeneity (Jiang & Zuo, 2016), their higher reliability indicates that the extent of specialization may be generally consistent during jhana meditation.

Although there are several other areas demonstrating good ReHo reliability across jhanas, some of which likely subserve vital aspects of jhana phenomenology (e.g., attentional monitoring by DLFPC [Friehs et al., 2020; MacDonald et al., 2000], alterations in perception and awareness by thalamus [Hwang et al., 2017; Müller et al., 2023]), further multi‐subject neuroimaging studies of jhana meditation are necessary to definitively illuminate the functional validity of all the reliable brain networks/groups found here.

### 4.2 Effect of phenomenology and variations in reliability across individual jhanas

ReHo of some brain networks/groups demonstrated good reliability in specific but not all jhanas. In some cases, the sources of run‐to‐run variability in ReHo could be attributed to various phenomenological factors (i.e., attentional depth, attentional width, and intensity of jhana) that likely influenced the quality and experience of each jhana.

For instance, Yang et al. (2023) observed that ReHo values of specific brain areas (e.g., visual, parietal, DLPFC, mPFC, insula, and OFC areas) were significantly associated with self‐reported attentional stability, attentional width, and intensity of jhana. Consistent with these observations, we found that controlling for run‐to‐run variability in these phenomenological factors substantially improved reliability in several brain networks/groups (i.e., by decreasing run‐to‐run ReHo variability). Specifically, reliability of various visual, dorsal, and salience ventral attentional (comprising insular, pre‐ and post‐central, opercular, frontal, OFC areas), control (comprising temporal, parietal, lateral PFC, ACC areas), and default‐mode (mPFC, PCC, precuneus, temporal, parahippocampus, retrosplenial areas) network regions surpassed the threshold in several jhanas only after controlling for the inter‐run variance in these phenomenological attributes. These networks also demonstrated modest functional integration (average ReHo ≥ 0.1) in multiple jhanas. Phenomenological data sampling concurrent to neuronal measurements can hence be valuable in better characterizing and explaining neuronal dynamics underlying complex states of consciousness (Timmermann et al., 2023), as well as effectively complementing existing nuanced phenomenological classifications of meditative states and traits (Sparby & Sacchet, 2022). Furthermore, controlling for head motion artifacts also appeared to improve the reliability estimates of few networks/groups, albeit minimally. Therefore, it is important to consider and adequately control for these artifactual sources of variation during future neuroimaging analyses of jhana meditation, as some networks/groups may be slightly more related to head motion than others (e.g., certain limbic and control network areas surpassing reliability threshold only after mFD regression).

Among subcortical regions, after thalamus, the hippocampus demonstrated the most replicable engagement with jhana meditation regardless of phenomenological variance, particularly during the form jhanas (e.g., J1, J2, and J3). These regions may thereby support specific aspects of memory, cognitive, and perceptual processes integral to the earlier jhanas. Reliability associated with cerebellar and brainstem areas did not surpass the threshold in any jhana, even after accounting for variance in attentional properties and intensity of jhana. Involvement of these areas in jhanas may not be as consistent, or may be partly influenced by variability in other state‐specific phenomenological elements (e.g., joy in J2, equanimity in J4, formlessness in J5–J8) (Yang et al., 2023) or efficiency of neuronal dynamics (Brefczynski‐Lewis et al., 2007; Escrichs et al., 2019; Hiroyasu & Hiwa, 2017). Incorporating detailed phenomenological reports with brain function modeling (i.e., neurophenomenology) can potentially accentuate the sources of unexplained variability associated with neural dynamic modeling of advanced meditative states (Timmermann et al., 2023).

Finally, we also found noticeable hemispheric (left–right) asymmetries in brain network ICC values within most of the brain networks/groups examined, such that some areas exhibited good reliability in exclusively one (e.g., right thalamus in all jhanas) or the other (e.g., left primary somatomotor network in all jhanas) hemisphere. Future neuroimaging investigations are likely to benefit by closely considering the potential roles of each brain network's hemispheric divisions in jhana and advanced meditation, given large‐scale meta‐analytic evidence suggesting general functionally relevant asymmetries in cortical (Kong et al., 2018) and subcortical (Guadalupe et al., 2017) regions.

### 4.3 Utility of brain network ICC

Within‐subject fMRI reliability estimates can provide preliminary brain templates for further detailed and larger neuroimaging investigations of rare conditions or states such as jhanas. Within the framework of meditation practice, examining the consistency and reliability of brain responses under repeated runs of instructed meditative states using brain network ICC could facilitate objective benchmarking of meditative development (Galante et al., 2023; Wright et al., 2023). Furthermore, brain network ICC maps of the most reliable brain areas in specific jhanas can efficiently inform the future development of advanced multivariate jhana decoders that can utilize subtle neurophenomenological elements of distinct jhanas, through sophisticated machine learning methodologies including multivariate voxel pattern analysis (Norman et al., 2006), multi‐timepoint pattern analysis (Ganesan, Lv, & Zalesky, 2022), etc. Consequently, such multivariate decoders can enable neurobiological monitoring of jhanas via neuromodulation and neurofeedback devices, which can thereby potentially improve the accessibility of jhanas and their benefits to the wider population regardless of meditation expertise.

Brain network ICC can also be implemented in multi‐subject longitudinal studies, by performing traditional group statistics (e.g., general linear modeling) across subject‐level brain network ICC estimates. Brain networks with above‐threshold reliability in specific jhanas likely demonstrate stable associations with those jhanas, which can also improve the statistical likelihood of detecting effects in these networks with small samples (Zuo et al., 2019). However, note that high reliability alone does not necessarily imply strong functional relevance of observed brain responses (Noble et al., 2019, 2021).

### 4.4 Limitations

The findings from this intensively sampled single‐subject case study need to be interpreted considering several limitations. First, although the experimental design involves numerous repeated measurements (i.e., 27 fMRI runs) which can promote internal replicability and validity, the generalizability of these findings is nevertheless limited due to reliance on single subject (*N* = 1) data. Note that the proposed brain network ICC approach is intended for within‐subject fMRI reliability analysis; however, it can also be extended in the future to make group‐level inferences by summarizing across subject‐level ICC maps. There is however a need for future validation of brain network ICC as a robust within‐subject fMRI reliability tool using other samples and conditions. Specifically, the findings presented here using 7 T fMRI, although functionally specific to jhanas, need to be interpreted with caution, until further broader characterizations of longitudinal variations in ReHo pertaining to widely studied fMRI contexts (e.g., resting‐state, motor task, memory task) are possible. This also highlights the need for the broader neuroimaging community to consider acquiring more longitudinal fMRI datasets to enable rigorous evaluations of within‐subject fMRI reliability. Second, ICC methods generally rely on intra‐ as well as inter‐individual variability, such that high ICC values may not necessarily guarantee high replicability, since they can also result from higher heterogeneity across individuals (or across regions within brain network in the case of brain network ICC). However, the likelihood of ICC estimates being exclusively driven by intra‐network heterogeneity may be low, since variability between constituent regions of brain networks/groups (average relative standard deviation of normalized ReHo across constituent regions) did not show any significant association with the brain network ICC estimates. Finally, there are different substyles of jhana meditation practiced around the world (e.g., kasina practice, mindful breathing) (Shankman, 2008a) and therefore the reliability of brain areas may change with the practice substyle.

### 4.5 Conclusion

For the first time, we assessed the reliability of fMRI ReHo associated with a single subject (*N* = 1) sampled intensively (27 fMRI runs over 5 days) using ultrahigh field 7 Tesla fMRI during advanced jhana meditation. To accomplish this, we proposed a modified intraclass correlation (ICC) approach called brain network ICC to examine within‐subject fMRI reliability at the level of canonical brain networks. We found several brain networks/groups with modest ReHo and good reliability across distinct jhanas spanning somatomotor, limbic, default‐mode, control, temporoparietal, and thalamic areas. These networks and constituent regions potentially underpin core neurocognitive mechanisms of jhana meditation.

Additionally, we found that the reliability of ReHo in some networks/groups was below‐threshold in specific but not all jhanas. However, on accounting for variability in self‐reported phenomenological factors such as attentional depth, attentional width, and jhana intensity, most of these networks/groups improved reliability and crossed the threshold across jhanas. Comparatively, the impact of head motion on overall reliability estimates was fairly minimal; however, future studies should adequately consider the influence of head motion artifacts while investigating neural correlates of jhanas. Overall, our findings provide a preliminary template of brain networks to contextualize prior results from neuroimaging of jhana meditation, as well as inform the design of future larger experiments. We recommend implementing rigorous neurophenomenological approaches while investigating advanced meditation to effectively capture the most robust brain mechanisms and subtle dynamics underlying advanced states of consciousness, such as jhanas.

## AUTHOR CONTRIBUTIONS

SG was involved in computational methods conceptualization, analysis, and manuscript writing and revision; WFZY was involved in data preprocessing, supervision, and manuscript revision; AC was involved in data acquisition, data preprocessing, and manuscript revision; AZ was involved in computational methods conceptualization, supervision, and manuscript revision; MDS was involved in study conceptualization and design, supervision, and manuscript revision.

## FUNDING INFORMATION

MDS and the Meditation Research Program (including WFZY and AC) are supported by the National Institute of Mental Health (Project Number R01MH125850), Dimension Giving Fund, Ad Astra Chandaria Foundation, Brain and Behavior Research Foundation (grant number 28972), BIAL Foundation (grant number 099/2020), and individual donors. SG is supported by Australian Research Training Program scholarship and Graeme Clark Institute Top‐up scholarship. AZ is supported by National Health and Medical Research Council Senior Research fellowship (APP1118153) and the Rebecca L. Cooper Medical Research Foundation.

## CONFLICT OF INTEREST STATEMENT

The authors declare no conflicts of interest.

## Supporting information


**Data S1.** Supporting information.

## Data Availability

The data that support the findings will be available upon reasonable request.
