# Supplementary material for: Within‐subject reliability of brain networks during advanced meditation: An intensively sampled 7 Tesla MRI case study
Source: Hum Brain Mapp. 2024 May 10;45(7):e26666. doi: 10.1002/hbm.26666 (PMC11082832; doi:10.1002/hbm.26666)
Supplement: Supplementary file 1 — Data S1. Supporting information. [file HBM-45-e26666-s001.docx]

Supplementary Material

Within-subject reliability of brain networks during advanced meditation: An intensively sampled 7 Tesla MRI case-study

**Supplementary section SS1: Background on ICC**

Traditionally, ICC is defined as the proportion of total variability in measurements (e.g., across sessions and subjects) that is attributed to variability between subjects. Higher ICC values indicate that most of the total variability is due to variability between subjects, and that variability within subjects (i.e., between repeated measurements) is comparatively smaller (i.e., more reliable). On the contrary, lower ICC values indicate lower reliability (and higher variability) of repeated measurements. ICC values vary between 0 and 1, and can be qualitatively classified as “poor” (ICC < 0.5), "moderate" (0.5–0.75), “good” (0.75–0.9) and "excellent" (ICC > 0.9) (Koo & Li, 2016; Liljequist et al., 2019). In the context of fMRI, ICC is typically used to quantify reliability of repeated fMRI measurements (i.e., runs/sessions) in samples comprising multiple subjects (N>1) (Noble et al., 2021). Mathematically, ICC is broadly formulated as

$ICC =\frac{\sigma_{s}}{\sigma_{s}+\sigma_{r}}$ (1)

where $\sigma_{s}$ refers to between-subject variance and $\sigma_{r}$ refers to within-subject (or between-run) variance. The denominator in (1) thus represents the total variance across runs and subjects.

The above-mentioned subjects in traditional ICC would be replaced by brain regions within a network/group in brain network ICC. In other words, for each brain network/group, brain network ICC is estimated by calculating the proportion of total variance of ReHo (across runs and regions) that is attributed to variance of ReHo between regions constituting the brain network/group.

**Supplementary section SS2: Model, definition and type of brain network ICC**

Traditionally, there are several forms of ICC based on *model*, *type* and *definition* (see Koo and Li (2016) for details). To calculate brain network ICC for within-subject reliability, we used the standard mathematical formulation of ICC(A,1) (also referred to as ICC(2,1)) with 2-way mixed effects *model*, “absolute agreement” *type*, and “single-rater” *definition* (Chen et al., 2018; Liljequist et al., 2019).

For a given subject, brain network ICC estimates would be generalizable when fMRI runs are sampled randomly from a larger distribution of fMRI runs associated with a given state (e.g., specific jhana) of the subject. On the other hand, brain regions would be fixed for a given brain network/group of a subject. Therefore, the most suitable model to estimate within-subject brain network ICC is the 2-way mixed effects *model*, where regions are considered fixed (i.e., fixed effects) and runs random (i.e., random effects).

For *type* and *definition* of brain network ICC, we adopted “single-rater” and “absolute agreement” options respectively. Compared to the “consistency” *definition* and “mean of k raters” *type*, “absolute agreement” and “single rater” are more conservative and produce smaller ICC estimates (Koo & Li, 2016). This is because absolute agreement requires the repeated measures to agree with each other, while “consistency” merely concerns the relative ordering of values under each repeated measurement. Additionally, ICC with the “consistency” definition does not explicitly model random measurement biases, which could potentially inflate ICC estimates in the presence of unknown measurement biases (Liljequist et al., 2019). Similarly, “single rater” type involves comparisons with individual instances of repeated measurements, while “mean of k raters” compares with averages across all the repeated measures. Furthermore, thresholding of brain network ICC values (≥ 0.75) was performed on the lower limit of their 95% confidence intervals, thereby minimizing the likelihood of inflated ICC estimates.

Overall, due to these conservative settings, we expect the brain network ICC estimates to be fairly reliable, as they would sufficiently account for measurement biases and errors across runs.. To calculate brain network ICC, we used standard mathematical formulation of ICC(A,1) which satisfies all the aforementioned criteria, i.e., 2-way mixed effects *model*, “absolute agreement”, and “single-rater”. Formal mathematical descriptions of ICC(A,1) can be found in McGraw and Wong (1996) and Liljequist et al. (2019). Note that ICC(A,1) is alternatively also referred to as ICC(2,1) (Chen et al., 2018), and their mathematical formulations and interpretations are identical.

***Figure S1:*** *Brain network ICC values mapped onto the brain surface (cortex) and discrete brain volume slices (subcortex, cerebellum and brainstem) for sequential jhanas J1 to J8, estimated from region-level ReHo values. A) Brain network ICC visualization of brain networks/groups with at least “good” lower confidence limits (ICC lower limit >= 0.75), where the ICC value of each brain network/group determines their color (“hot” color gradient). Note that jhanas J6-J8 were merged as one advanced state during analyses. B) Same as (A) but after controlling region-level ReHo values for key self-report phenomenological variables (1 value per fMRI run per jhana) including “Attentional stability”, “Attentional width”, and “jhana intensity”. ICC - Intraclass Coefficient Correlation, S – superior/dorsal, A – anterior, P – posterior, I – inferior/ventral, L – Left, R – Right.*

***Figure S2:*** *Average ReHo values of brain networks/groups with above-threshold reliability mapped onto the brain surface (cortex) and discrete brain volume slices (subcortex, cerebellum and brainstem) for sequential jhanas J1 to J8. A) Visualization of ReHo values averaged across runs and regions within each brain network/group that demonstrated above-threshold brain network ICC. The average ReHo of each brain network/group determines their color (“spectral” color gradient). Note that jhanas J6-J8 were merged as one advanced state during analyses. B) Same as (A) but the brain networks/groups with above-threshold reliability were characterized by estimating ICC after controlling region-level ReHo values for key self-report phenomenological variables (1 value per fMRI run per jhana) including “Attentional stability”, “Attentional width”, and “jhana intensity”. Note that the average ReHo values are the same for (A) and (B), but the networks/groups with above-threshold reliability may be different (as shown in Figure S1). ICC - Intraclass Coefficient Correlation, S – superior/dorsal, A – anterior, P – posterior, I – inferior/ventral, L – Left, R – Right.*

***Figure S3:*** *Graphical representations of changes in the lower confidence limits of brain network ICC values that were above-threshold across all the jhanas J1-J8, after controlling the region-level ReHo values for (A) key self-report phenomenological variables (1 value per fMRI run per jhana), i.e., “Attentional stability”, “Attentional width”, and “jhana intensity”, and (B) the self-report phenomenological variables as well as mean framewise displacement (mFD = 1 value per fMRI run per jhana representing head motion artifact). The circled network in (A) had below-threshold ICC in one of the jhanas only after controlling for mFD. Note that jhanas J6-J8 were merged as one advanced state. ICC change of only the labelled network/group is highlighted (black line) in each graph while the remaining networks/groups are greyed out in the background. The y-axis of each graph represents brain network ICC values while the distinct jhanas (J1 to J6-J8) are marked on the x-axes. The dotted red line in each graph represents the threshold of “good” brain network ICC (i.e., ICC lower confidence limit of 0.75). ICC - Intraclass Coefficient Correlation, ReHo - Regional Homogeneity, A – anterior, P – posterior, I – inferior/ventral, L – Left, R – Right.*

***Figure S4:*** *Graphical representations of changes in the lower confidence limits of brain network ICC values that were above-threshold in at most 5 of the 6 jhanas (where jhanas J6-J8 are merged), after controlling the region-level ReHo values for (A) key self-report phenomenological variables (1 value per fMRI run per jhana), i.e., “Attentional stability”, “Attentional width”, and “jhana intensity”, and (B) the self-report phenomenological variables as well as mean framewise displacement (mFD = 1 value per fMRI run per jhana representing head motion artifact). The circled networks in (B) had above-threshold ICC in at least one of the jhanas only after controlling for mFD. Note that ICC change of only the labelled network/group is highlighted (black line) in each graph while the remaining networks/groups are greyed out in the background. The y-axis of each graph represents brain network ICC values while the distinct jhanas (J1 to J6-J8) are marked on the x-axes. The dotted red line in each graph represents the threshold of “good” brain network ICC (i.e., ICC lower confidence limit of 0.75). The green coloured points indicate above-threshold ICC (i.e., ICC lower confidence limit ≥ 0.75) while red coloured points indicate below-threshold ICC (i.e., ICC lower confidence limit < 0.75) for a given jhana. ICC - Intraclass Coefficient Correlation, ReHo - Regional Homogeneity, A – anterior, P – posterior, I – inferior/ventral, L – Left, R – Right.*

**Supplementary Table S1:** List of all the brain regions constituting each cortical brain network (based on Yeo cortical network atlas) and each group of brain areas within the subcortex, cerebellum and brainstem. Each network/group label listed here was split into left (L) and right (R) sub-networks/sub-groups wherever feasible. The total number of networks/groups used for analyses after the hemispheric splitting where applicable is 50.

|  | **Brain network/group label** | **Constituent brain regions (with number of sub-divisions in each hemisphere)** |
| --- | --- | --- |
| 1 | Central Visual | striate cortex (1L, 1R), extra-striate cortex (11L, 11R) |
| 2 | Peripheral Visual | striate calcarine (2L, 2R), extra-striate inferior (5L, 5R), extra-striate superior (5L, 4R) |
| 3 | Somatomotor A | somatomotor A (19L, 20R) |
| 4 | Somatomotor B | S2 (6L, 8R), insula (1L, 1R), auditory (4L, 3R), somatomotor central (5L, 3R) |
| 5 | Dorsal Attention A | temporal occipital (4L, 3R), parietal occipital (2L, 3R), superior parietal lobule (7L, 8R) |
| 6 | Dorsal Attention B | post central (9L, 8R), frontal eye fields (3L, 3R), precentral ventral (1L), temporal occipital (1R) |
| 7 | Salience Ventral Attention A | parietal operculum (3L, 3R), frontal operculum (2L, 3R), insula (4L, 4R), parietal medial (3L, 4R), frontal medial (3L, 4R), precentral (1R) |
| 8 | Salience Ventral Attention B | lateral prefrontal cortex (3L, 3R), insula (3L, 2R), orbital frontal cortex (1L), medial posterior prefrontal cortex (1L, 2R), inferior parietal lobule (1R), lateral ventral prefrontal cortex (1R) |
| 9 | Limbic A | temporal pole (7L, 6R) |
| 10 | Limbic B | orbital frontal cortex (5L, 6R) |
| 11 | Control A | temporal (1L, 1R), intraparietal sulcus (5L, 4R), dorsal prefrontal cortex (1L, 1R), lateral prefrontal cortex (3L, 5R), lateral ventral prefrontal cortex (2L), mid-cingulate (1L, 1R) |
| 12 | Control B | temporal (2L, 2R), inferior parietal lobule (3L, 4R), dorsal prefrontal cortex (1L, 1R), dorsolateral prefrontal cortex (4R), lateral ventral prefrontal cortex (3L, 4R), medial posterior prefrontal cortex (1L, 1R) |
| 13 | Control C | precuneus (3L, 5R), posterior cingulate (2L, 2R) |
| 14 | Default-mode A | inferior parietal lobule (2L, 2R), dorsal prefrontal cortex (3L, 2R), precuneus-posterior cingulate cortex (7L, 5R), medial prefrontal cortex (6L, 6R), temporal (1R) |
| 15 | Default-mode B | temporal (6L, 2R), inferior parietal lobule (2L), dorsal prefrontal cortex (6L, 5R), lateral prefrontal cortex (2), ventral prefrontal cortex (5L, 3R), anterior temporal (1R) |
| 16 | Default-mode C | inferior parietal lobule (1L, 2R), retrosplenial cortex (3L, 2R), parahippocampal cortex (3L, 2R) |
| 17 | Temporoparietal | temporal parietal (6L, 10R) |
| 18 | Thalamus | anterior thalamus (5L, 5R), posterior thalamus (4L, 4R) |
| 19 | Hippocampus | hippocampal head (4L, 4R), hippocampal body (2L, 2R), hippocampal tail (1L, 1R) |
| 20 | Caudate + Putamen | anterior caudate (3L, 3R), posterior caudate (1L, 1R), anterior putamen (2L, 2R), posterior putamen (2L, 2R) |
| 21 | Amygdala + Globus Pallidus + Nucleus Accumbens | lateral amygdala (2L, 2R), superior medial amygdala (1L, 1R), anterior globus pallidus (1L, 1R), posterior globus pallidus (1L, 1R), nucleus accumbens core (1L, 1R), nucleus accumbens (1L, 1R) |
| 22 | Brainstem raphe areas | caudal-rostral linear raphe, dorsal raphe, medial raphe, paramedian raphe, raphe magnus, raphe obscurus, raphe pallidus |
| 23 | Brainstem nuclei complex | cuneiform nucleus (1L, 1R), inferior olivary nucleus (1L, 1R), laterodorsal tegmental nucleus (1L, 1R), lateral parabrachial nucleus (1L, 1R), parabigeminal nucleus (1L, 1R), medial parabrachial nucleus (1L, 1R), pedunculotegmental nucleus (1L, 1R), red nuclei (2L, 2R), viscero-sensory-motor nuclei complex (1L, 1R), vestibular nuclei complex (1L, 1R) |
| 24 | Brainstem reticular areas | inferior medullary reticular formation (2L, 2R), isthmic reticular formation (1L, 1R), mesencephalic reticular formation (3L, 3R), parvicellular reticular formation (1L, 1R), pontine reticular nucleus (1L, 1R), superior medullary reticular formation (2L, 2R) |
| 25 | Brainstem non-nuclei areas | inferior colliculus (1L, 1R), locus coeruleus (1L, 1R), periaqueductal gray, superior colliculus (1L, 1R), superior olivary complex (1L, 1R), substantia nigra (2L, 2R), subcoerulus (1L, 1R) |
| 26 | Cerebellum | cerebellar areas (10 from MDTB atlas) |

L – left, R – Right, MDTB - Multi-Domain Task Battery, number preceding L and R indicates the number of sub-regions in the left and right hemisphere of the given region

**References**

Chen, G., Taylor, P. A., Haller, S. P., Kircanski, K., Stoddard, J., Pine, D. S., . . . Cox, R. W. (2018). Intraclass correlation: Improved modeling approaches and applications for neuroimaging. *Hum Brain Mapp, 39*(3), 1187-1206. doi:10.1002/hbm.23909

Koo, T. K., & Li, M. Y. (2016). A Guideline of Selecting and Reporting Intraclass Correlation Coefficients for Reliability Research. *J Chiropr Med, 15*(2), 155-163. doi:10.1016/j.jcm.2016.02.012

Liljequist, D., Elfving, B., & Skavberg Roaldsen, K. (2019). Intraclass correlation - A discussion and demonstration of basic features. *PLoS One, 14*(7), e0219854. doi:10.1371/journal.pone.0219854

McGraw, K. O., & Wong, S. P. (1996). Forming inferences about some intraclass correlation coefficients. *Psychological Methods, 1*, 30-46. doi:10.1037/1082-989X.1.1.30

Noble, S., Scheinost, D., & Constable, R. T. (2021). A guide to the measurement and interpretation of fMRI test-retest reliability. *Curr Opin Behav Sci, 40*, 27-32. doi:10.1016/j.cobeha.2020.12.012
